# Executive Function and the P300 after Treadmill Exercise and Futsal in College Soccer Players

**DOI:** 10.3390/sports5040073

**Published:** 2017-09-26

**Authors:** Junyeon Won, Shanshan Wu, Hongqing Ji, J. Carson Smith, Jungjun Park

**Affiliations:** 1 Division of Sport Science, Pusan National University, Busan 46241, Korea; won25@umd.edu (J.W.); wushanshan521533@gmail.com (S.W.); jihongqing0825@gmail.com (H.J.); 2 Department of Kinesiology, University of Maryland, College Park, MD 27042, USA; carson@umd.edu

**Keywords:** event-related potential, P300, exercise mode, athletes, soccer, executive function, EEG, cognition

## Abstract

**(1) Background:** Although a body of evidence demonstrates that acute exercise improves executive function, few studies have compared more complex, laboratory-based modes of exercise, such as soccer that involve multiple aspects of the environment. **(2) Methods:** Twelve experienced soccer players (24.8 ± 2 years) completed three counterbalanced 20 min sessions of (1) seated rest; (2) moderate intensity treadmill exercise; and (3) a game of futsal. Once heart rate returned to within 10% of pre-activity levels, participants completed the Stroop Color Word Conflict Task while reaction time (RT) and P300 event-related potentials were measured. **(3) Results:** Reaction time during Stroop performance was significantly faster following the futsal game and treadmill exercise compared to the seated rest. The P300 amplitude during Stroop performance was significantly greater following futsal relative to both treadmill and seated-rest conditions. **(4) Conclusions:** These findings suggest that single bouts of indoor soccer among college-aged soccer players, compared to treadmill and seated-rest conditions, may engender the greatest effect on brain networks controlling attention allocation and classification speed during the performance of an inhibitory control task. Future research is needed to determine if cognitively engaging forms of aerobic exercise may differentially impact executive control processes in less experienced and older adult participants.

## 1. Introduction

A body of literature has demonstrated that exercise exerts positive impacts on cognitive performance [1,2,3]. Similarly, a wide range of research in humans and animals indicates that aerobic exercise is closely associated with enhancement of cognitive function [4,5]. In particular, aerobic exercise has been shown to exert particularly large effects on working memory [6] and executive function [7]. However, most studies have used a simple mode of acute exercise, such as treadmill running, and few have examined the effects of exercise that involve greater degrees of working memory load and executive function.

The effects of a single bout of exercise on cognitive performance have been observed in previous studies. Sibely and colleagues [8] observed facilitations in cognitive performance through a Stroop task that took place immediately after a 20 min bout of aerobic exercise. Another study found that, following a 30 min bout of exercise, participants’ performance on the Paced Auditory Serial Addition Test improved [9]. Collectively, these investigations regarding behavioral performance suggest that a single bout of exercise improves indices of inhibitory control [10].

Beyond the behavioral indices, the examination of electroencephalographic (EEG) activity provides an indicator of specific cognitive operations. Several lines of research [11,12] have reported effects of acute exercise on the P300 component of the event-related potential (ERP). Approximately 300–800 ms following the presentation of a stimulus, the P300, a peak-positive component of the ERP, can be observed. The amplitude of P300 represents, in part, the amount of attentional resources allocated toward the stimulus environment, while its latency is used as an index of stimulus classification speed [13]. Past acute exercise studies have found increased P300 amplitude and decreased P300 latency following single bouts of aerobic exercise. Specifically, after a 30 min bout of exercise, an increase in P300 amplitude was observed during all phases of flanker task performance, while a reduction in P300 latency was observed during the incongruent trials that require greater amounts of inhibitory control [14,15].

Among the studies that have reported positive effects of acute exercise on cognitive function, most have used treadmills [16,17] or cycle ergometer exercise [18,19]. Only a handful of investigations have assessed the difference between modes of exercise on indices of executive control. O’Leary et al. [20] compared the effects of “exergames” and treadmill-based exercise on executive control and found faster RT and increased P300 amplitude following treadmill exercise relative to seated rest and videogame play. They concluded that, since the participants were not familiar with the dynamic nature of videogame play, the unfamiliarity negated beneficial effects on cognitive function elicited by videogame play.

The purpose of this study was to assess the effects of acute futsal (indoor soccer), compared to acute treadmill exercise and a no-exercise control condition, among experienced soccer players on reaction time and event-related potentials during the performance of the Stroop Color Word Conflict task. Based on previous evidence [20], we hypothesized that reaction time and P300 amplitude of soccer players following a futsal game would improve relative to rest and treadmill exercise, because it was thought that experiencing the dynamic nature of the exercise would lead to reduced reaction time and greater P300 amplitude.

## 2. Materials and Methods

### 2.1. Participants

A priori power analysis indicated that a sample size of 12 would be sufficient to detect a significant effect with a power of 0.9 and the standardized effect size of 1.4. Twelve male soccer players were recruited from the soccer team of Pusan National University, South Korea. All participants provided written informed consent approved by the Institution Review Board at Pusan National University. All participants were not taking neurological medication, and their medical history was free of neurological problems. Participants were asked to abstain from physical activity on the days they engaged in the experiment. The participants were also instructed to eat usual meals and to finish meals 4 h before the experiment, to avoid alcohol 1 day before the experiment, and to avoid caffeine in the 4 h prior to participation each day [21]. Demographic and fitness data for all participants are provided in Table 1. This study was conducted according to the Helsinki Declaration of 1975 [22].

### 2.2. Procedure

This experiment included 3 different conditions; seated rest, treadmill exercise, and futsal. Each condition was completed by each participant, conducted on different days, with an interval of 7 days between conditions in an effort to minimize familiarization with the cognitive task, as reported in a previous study [23]. For seated rest, subjects were seated comfortably before performing the Stroop task. Following each condition, participants were prepared for EEG measurement to assess P300 amplitude and RT. After the treadmill exercise and futsal conditions, cognitive testing was performed once participants’ HR returned to within 10% of pre-exercise levels.

### 2.3. Exercise Task

Ratings of perceived exertion (RPE) and heart rate (HR) were used to measure the intensity of the exercise conditions. HR and RPE were recorded every 5 min. RPE values were recorded with the Borg 6–20 RPE scale [24]. Using a within-subject design, the order of the experimental conditions (treadmill and futsal) was counterbalanced across participants to reduce the possibility of learning or habituation effects. Prior to each session, participants were fitted with a Heart Rate Monitor (H1 HR Sensor, Polar Electro, Kempele, Finland). The treadmill exercise and futsal session each lasted 30 min, during which time HR and RPE were assessed every 5 min. During the futsal session, participants played in a 6 on 6 game within a court 15 m × 25 m, the minimum size of a standard official futsal court [25]. The court size was selected to result in moderate intensity exercise (60–65% HR_max_ and RPE at 12–14). Although Foster 0–10 scale [26] is normally used to measure perceived exertion of professional soccer [27,28], in order to match exercise intensity with the treadmill, Borg 6–20 scale was used in this study. During each game, if a participant’s HR was at or below the targeted level, they were instructed to perform actively. On the other hand, if a participant’s HR was above the target HR, they were encouraged to perform less actively. During the treadmill exercise, participants ran on a motor-driven treadmill at 65% HR_max_ and an RPE of 12–14 for 20 min. The futsal games took place on 12 different days, among the same 12 players, with a three-day inter-game interval. After each game, one player underwent EEG recordings and performed the Stroop task.

### 2.4. Stroop Task

Subjects were seated 1 m from the computer screen with a keyboard resting on a desk. They were instructed to perform the standard Stroop color-naming task, in which only the display color of the word was relevant [28]. They were told to use their right index, or right middle fingers to indicate whether the stimulus word was presented in red, green, blue, or yellow. To discourage eye movements, participants were constantly instructed to look ahead at the monitor rather than down at their fingers during task performance. Subjects underwent practice of pressing the response buttons until they were ready to start the test [28]. The number of practice trials was standardized (2 trials each) so that participants performed the same number of practice trials before each condition. Telescan software (LAXTHA, Daejeon, South Korea) was used for the Stroop task, in which participants were instructed to respond as quickly as possible. Stimulus duration was 100 ms, and the inter-stimulus interval was varied randomly between 2000 and 2500 ms for minimized expectation effects [28]. Each block consisted of 60 trials, with stimulus probability equated across the 4 display color conditions and 4 congruent trials.

### 2.5. Electroencephalography Measurements

According to the international 10–20 system, electroencephalographic (EEG) activity was recorded with Ag–AgCl electrodes at frontal (Fz), central (Cz), and parietal (Pz) midline locations referenced to linked electrodes placed on the left and right earlobes, with a forehead ground using WEEG-32 (LAXTHA, Daejeon, South Korea). EEG activity was recorded with electrodes attached with paste and tape at each sites. All electrodes had a resistance of less than 5 kΩ. Event-related potential (ERP) component amplitudes were assessed with a computer-assisted peak detecting system, with peak amplitude ascertained manually when the program failed to identify a peak. After completing the experiment, EEG signals were analyzed with software (Telescan, LAXTHA, South Korea). ERPs time-locked to the target stimulus were extracted using ensemble averaging (450 Hz sampling rate). The P300 component was defined as the largest positive peak occurring between 300 and 700 ms. P300 amplitude was measured as the difference between the mean pre-stimulus baseline and maximum peak amplitude. Telescan’s built-in high pass IIR filter was used for filtering. Waveforms were digitally smoothed with a low-pass filter using a half-power cutoff of 10 Hz prior to analysis.

### 2.6. Statistical Analysis

One-way repeated measures ANOVA was used to determine significant differences in HR, RPE, RT, and P300 amplitude between the three conditions (rest, treadmill exercise, and futsal). Mauchly’s test of sphericity was computed for each ANOVA; however, there were no violations of the sphericity assumption, so the results were reported using unadjusted degrees of freedom. Paired *t*-tests were used for post-hoc comparisons, and the False Discovery Rate was applied to adjust for family-wise error within each variable. Statistical significance was set at a two-tailed *p <* 0.05.

## 3. Results

### 3.1. Manipulation Checks—HR and RPE

Data from the heart rate monitor indicated the mean HR (±SD) for the seated rest, treadmill, and futsal conditions were 81.1 ± 3.7 bpm, 131.7 ± 2.1 bpm, and 133.1 ± 3.1 bpm, respectively (Condition main effect, F(2, 22) = 1308.3, *p <* 0.001, η^2^ = 0.992). HR was significantly greater in the treadmill exercise and futsal conditions compared to the seated rest condition (both *p <* 0.001), and there were no statistically significant differences in HR between the treadmill and futsal conditions (*p =* 0.184). Similarly, ratings of perceived exertion for the seated rest, treadmill, and futsal conditions were 6.0 ± 0.0, 12.5 ± 0.7, and 13.1 ± 0.6, respectively (Condition main effect, F(2, 22) = 520.9, *p <* 0.001, η^2^ = 0.979). The treadmill exercise and futsal conditions showed significantly greater RPE relative to the seated rest condition (both *p <* 0.001), and there were no statistically significant differences between the treadmill and futsal conditions (*p =* 0.089). The mean RPE value during the treadmill and futsal conditions was most closely associated with the verbal anchor “somewhat hard,” indicating moderate exercise intensity.

### 3.2. Response Time

**Figure 1:**
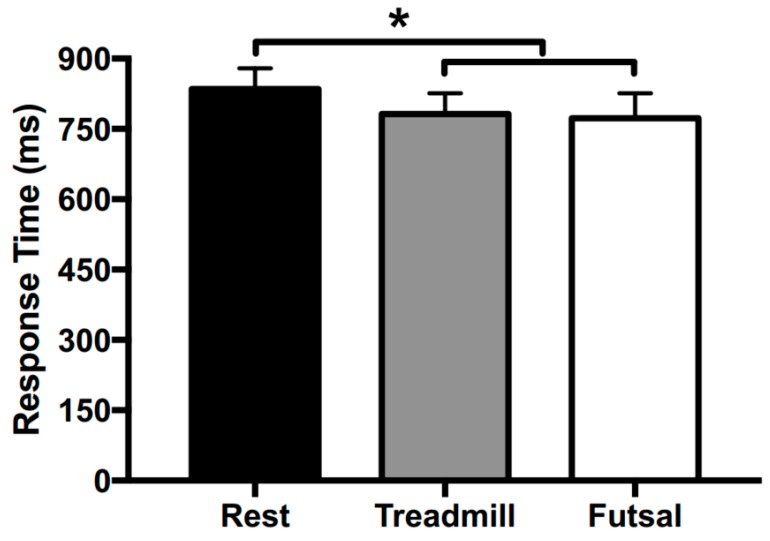
Mean response time (RT) during Stroop task performance after seated rest, treadmill exercise, and futsal. Error bars represent SEM. * Significant difference at *p <* 0.05.

During the performance of the Stroop task, the mean RT (±SD) was 835 ± 44 ms for seated rest conditions, 782 ± 44 ms for treadmill exercise conditions, and 773 ± 53 ms for futsal conditions (Condition main effect, F(2, 22) = 7.462, *p <* 0.005, η^2^ = 0.404; see Figure 1). RT was significantly faster under the treadmill exercise (*p =* 0.024) and futsal (*p =* 0.002) conditions compared to the rest condition, and there was no difference in RT between the treadmill exercise and futsal conditions (*p =* 0.566). 

### 3.3. Event-Related Potentials

**Figure 2:**
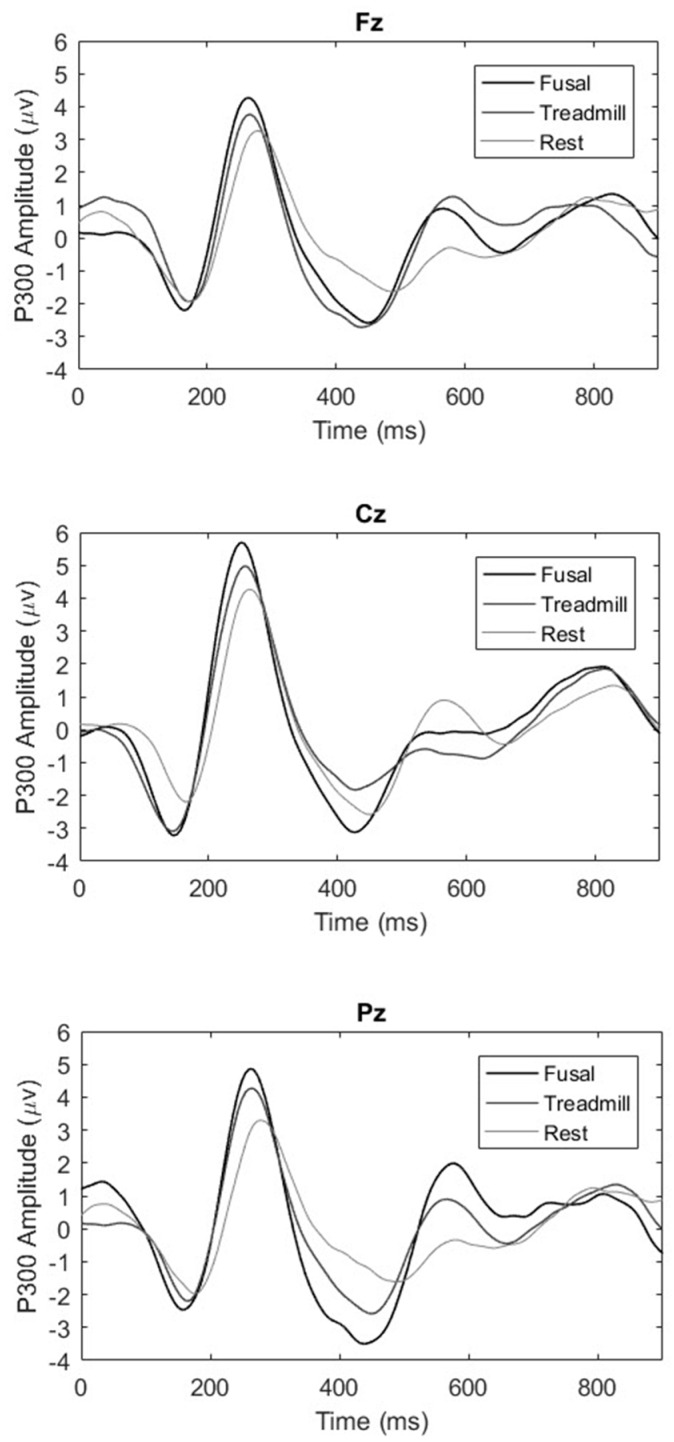
Mean P300 amplitudes from the Fz (**top panel**), Cz (**middle panel**), and Pz (**lower panel**) electrode sites during Stroop performance after seated rest (light grey), treadmill exercise (dark grey), and futsal (black).

The mean (±SD) P300 amplitudes for each condition (at each scalp recording site) were as follows: seated rest: Fz = 3.33 ± 0.19, Cz = 4.32 ± 0.21, Pz = 3.12 ± 0.16 μV; treadmill: Fz = 3.82 ± 0.15, Cz = 5.19 ± 0.58, Pz = 4.29 ± 0.68 μV); futsal: Fz = 4.01 ± 0.24, Cz = 5.6 ± 0.42, Pz = 4.73 ± 0.6 μV. At the frontal midline site (Fz), P300 amplitude was significantly greater after the treadmill (*p <* 0.00001) and futsal (*p <* 0.0001) compared to after seated rest, and was greater after futsal compared to treadmill exercise (*p =* 0.033) (Condition main effect, F(2, 22) = 34.024, *p <* 0.00001, η^2^ = 0.756). Results for the central midline site (Cz) also showed statistically higher P300 amplitude following treadmill exercise (*p =* 0.001) and futsal (*p <* 0.00001), compared to seated rest, and was greater after futsal compared to treadmill exercise (*p =* 0.037) (Condition main effect, F(2, 22) = 26.528, *p <* 0.00001, η^2^ = 0.707). Finally, following the same pattern at the parietal midline site (Pz), the P300 amplitude was significantly higher after the treadmill (*p =* 0.0001) and futsal (*p <* 0.00001) conditions, compared to seated rest, and, comparing treadmill and futsal conditions, the P300 amplitude was greater after futsal (*p =* 0.043) (Condition main effect, F(2, 22) = 38.803, *p <* 0.00001, η^2^ = 0.779). Each of the pairwise comparisons between conditions survived after correction for multiple comparisons using the false discovery rate. The mean ERP waveforms for each condition at each recording site are shown in Figure 2.

## 4. Discussion

In this study, the effects of single bouts of futsal and treadmill-based exercise on the cognition of soccer players were investigated using electroencephalographic and behavioral indices of cognitive testing. It was assumed that cognitive indices of soccer players after a futsal game, compared to seated rest and treadmill exercise, would show improvement, and this was based on the assumption that futsal may not be cognitively demanding for the subjects of this study, which may project results that are different from those of a previous study [20].

The current findings revealed enhanced P300 amplitude after playing a game of futsal relative to treadmill exercise and seated rest. The present findings suggest that cognitively challenging aerobic exercise engenders greater cognitive benefits for athletes than does exercise that is less demanding. The design of this study was set to hold the duration and intensity of exercise at a consistent level between treadmill exercise and futsal. The study protocol accomplished the goal with statistically no difference in HR between the two conditions, similar to previous findings of moderate aerobic activity [20]. Thus, the differences in P300 amplitude during the Stroop task between treadmill exercise and futsal cannot be explained by differences in cardiovascular or metabolic demand.

As a dynamic form of exercise, futsal requires several components of executive function. Players are constantly in need of anticipating movements of teammates and opponents before making decisions to where to pass or shoot. During this process, they need to recruit attentional focus over the course of 30 min of gameplay [29]. They also face new or changing environments on the field where the ability to gather relevant information in space and to recall game strategies are continually executed [30]. 

O’Leary and colleagues [20] reported faster RT and increased P300 amplitude during a control task following treadmill exercise relative to seated rest and an “exergaming” condition. However, as the authors of that study pointed out, the participants were not adept at the “exergame” (one practice session of the Wii™ game was provided before the experimental condition). As mentioned in O’Leary et al. [20], it is possible that the inexperience in the “exergaming” condition, which required greater attentional control to perform, may have been a source of increased executive control demand and stress, potentially offsetting the potential benefits of the exercise component. Considering their assumption, it might be that task proficiency (or lack thereof) rather than the exercise *per se* influenced the results reported by O’Leary et al. [20]. In the current study, both treadmill exercise and futsal resulted in greater P300 amplitude, with an added benefit for futsal over treadmill exercise, suggesting that there may have been a synergistic effect between the intensity of the exercise and the cognitive components required to play futsal. One caveat to this interpretation is that this pattern of results was observed for the P300 but not for response time, where there was an equally faster RT after both treadmill and futsal conditions.

Hockey [31] introduced a compensatory control model that includes the effects on performance during stressful conditions or high workload. This model suggested two negative feedback loops. The lower loop controls automatic processes, which explains why the maintenance of well-learned skills requires little effort. On the other hand, the upper loop manages the regulation of effort in stressful conditions and novel skills of an individual. This theory helps explain the results of the study by O’Leary et al. [20], where treadmill walking may be controlled by the lower loop, as it is an automated skill and performance requires little effort. Conversely, the exergaming condition may be cognitively more difficult since it was new to the participants who practiced it for a short period of time, and because a changing nature and variable attentional control is required for successful task completion. Paas et al. [32] also helps explain the disparity. It illustrates that the complexity of a task influences cognitive workload. Specifically, cognitively challenging exercise that requires bodily coordination demands additional cognitive effort. The more complicated the task, the more of an individual’s cognitive effort is required. Thus, complex exercise, compared to simple exercise, requires a greater cognitive workload. Considering the above theories, it would be difficult for participants with a short period of practice to play the Wii™ game, performing in a stressful upper-loop environment where a great deal of cognitive effort for body coordination is needed to accomplish the tasks. 

In the current study, we tested physically fit individuals, who were also very skilled at the task of playing soccer (most with greater than 10 years of soccer experience). The cardiovascular fitness combined with the skill and experience of the participants should result in relatively less effort learning the task of futsal. This suggests that our participants engaged in more of a “lower loop” automatic processes as opposed to an “upper-loop” environment [32]. Their task proficiency and skills at playing futsal may have provided a more autonomous exercise experience, and thus may have allowed more efficient engagement of executive control networks during play. In addition to the cognitive benefit from aerobic exercise, the constant engagement of soccer may help enhance attention and processing speed [33]. The dynamic nature of soccer games may help develop comprehensive cognitive benefits, which may be evidenced by improved behavioral performance and the engagement of attention networks, as shown by the ERP evidence.

Our findings of greater P300 amplitude after futsal compared to treadmill exercise and seated rest suggest that the cortical executive control networks were primed during futsal and that the ability to engage these networks was carried over into the post-game period. Therefore, it might be that, if individuals achieve high proficiency, cognitively demanding aerobic exercise may result in greater enhancement on cognitive control than exercise requiring less cognitive engagement.

There are several limitations of the current study. First, the participants consisted of only 12 male, fit, healthy, experienced athletes; thus, the homogeneous characteristics of the group, and the smaller sample size, limits the generalizability of the findings. Specifically, since the players were able to understand the complexity of the game based on their career, the activation on the brain might have been deeper and clearer compared to the general population, which makes it difficult to generalize the results of this study. The advantage to our approach, however, is that these participants were well skilled at playing futsal, and their similar background and demographic characteristics reduces variability that may be due to these factors. Second, only a single aspect of cognitive control was examined, specific to the Stroop task. Additional tasks should be investigated systematically in order to compare the effects of traditional forms of acute exercise to multi-player sport performance on executive function. Finally, due to a technical error, we were not able to separate the congruent and incongruent trial types of the Stroop task in our response time and ERP analyses. This limits the ability to interpret our findings only in terms of executive function. While the effects we observed after futsal and treadmill exercise might reflect aspects of executive control, it is also plausible that more general cognitive processes were affected, such as processing speed.

## 5. Conclusions

These findings suggest that single bouts of indoor soccer among college-aged soccer players, compared to treadmill exercise and seated-rest conditions, may produce the greatest effect on brain networks, controlling attention allocation and classification speed during the performance of an inhibitory control task. The result of this study advances our knowledge regarding the effect of the dynamic form of aerobic exercise on cognition as well as the significance of familiarity on the exercise format on cognitive performance. Future research is needed to determine if cognitively engaging forms of sport-related aerobic exercise may differentially impact executive control processes in less experienced and older adult participants. Another suggestion for future research is to include a control group of non-athlete subjects to see whether the results of the current study are applicable to general populations.

## Figures and Tables

**Table 1:** Mean (SD) values for participant demographic information.

Variable	All Participants (M ± SD)
Sample Size (*n*)	12
Sex	M
Age (years)	24.83 ± 2.08
Height (cm)	173.5 ± 4.96
Weight (kg)	68 ± 8.68
Soccer Career (years)	11.25 ± 2.34

## References

[1] Chaddock L., Hillman C.H., Pontifex M.B., Johnson C.R., Raine L.B., Kramer A.F. (2012). Childhood aerobic fitness predicts cognitive performance one year later. J. Sports Sci..

[2] Chang Y.-K., Tsai C.-L., Hung T.-M., So E.C., Chen F.-T., Etnier J.L. (2011). Effects of acute exercise on executive function: A study with a Tower of London Task. J. Sport Exerc. Psychol..

[3] Voss M.W., Chaddock L., Kim J.S., VanPatter M., Pontifex M.B., Raine L.B., Cohen N.J., Hillman C.H., Kramer A.F. (2011). Aerobic fitness is associated with greater efficiency of the network underlying cognitive control in preadolescent children. Neuroscience.

[4] Hamilton G.F., Rhodes J.S. (2015). Chapter Sixteen-Exercise Regulation of Cognitive Function and Neuroplasticity in the Healthy and Diseased Brain. Prog. Mol. Biol. Transl. Sci..

[5] Hillman C.H., Erickson K.I., Kramer A.F. (2008). Be smart, exercise your heart: Exercise effects on brain and cognition. Nat. Rev. Neurosci..

[6] Komiyama T., Sudo M., Higaki Y., Kiyonaga A., Tanaka H., Ando S. (2015). Does moderate hypoxia alter working memory and executive function during prolonged exercise?. Physiol. Behav..

[7] Chang Y.-K., Chu C.-H., Wang C.-C., Wang Y.-C., Song T.-F., Tsai C.-L., Etnier J.L. (2015). Dose-response relation between exercise duration and cognition. Med. Sci. Sports Exerc..

[8] Sibley B.A., Etnier J.L., Le Masurier G.C. (2006). Effects of an acute bout of exercise on cognitive aspects of Stroop performance. J. Sport Exerc. Psychol..

[9] Tomporowski P.D., Cureton K., Armstrong L.E., Kane G.M., Sparling P.B., Millard-Stafford M. (2005). Short-term effects of aerobic exercise on executive processes and emotional reactivity. Int. J. Sport Exerc. Psychol..

[10] Smith P.J., Blumenthal J.A., Hoffman B.M., Cooper H., Strauman T.A., Welsh-Bohmer K., Browndyke J.N., Sherwood A. (2010). Aerobic exercise and neurocognitive performance: A meta-analytic review of randomized controlled trials. Psychosom. Med..

[11] Hillman C.H., Snook E.M., Jerome G.J. (2003). Acute cardiovascular exercise and executive control function. Int. J. Psychophysiol..

[12] Kamijo K., Nishihira Y., Higashiura T., Kuroiwa K. (2007). The interactive effect of exercise intensity and task difficulty on human cognitive processing. Int. J. Psychophysiol..

[13] Polich J. (2007). Updating P300: An integrative theory of P3a and P3b. Clin. Neurophysiol..

[14] Brisswalter J., Collardeau M., René A. (2002). Effects of acute physical exercise characteristics on cognitive performance. Sports Med..

[15] Hillman C.H., Pontifex M.B., Raine L.B., Castelli D.M., Hall E.E., Kramer A.F. (2009). The effect of acute treadmill walking on cognitive control and academic achievement in preadolescent children. Neuroscience.

[16] Drollette E.S., Scudder M.R., Raine L.B., Moore R.D., Saliba B.J., Pontifex M.B., Hillman C.H. (2014). Acute exercise facilitates brain function and cognition in children who need it most: An ERP study of individual differences in inhibitory control capacity. Dev. Cogn. Neurosci..

[17] Schneider S., Brümmer V., Abel T., Askew C.D., Strüder H.K. (2009). Changes in brain cortical activity measured by EEG are related to individual exercise preferences. Physiol. Behav..

[18] Bailey S.P., Hall E.E., Folger S.E., Miller P.C. (2008). Changes in EEG during graded exercise on a recumbent cycle ergometer. J. Sports Sci. Med..

[19] Thacker J.S., Middleton L.E., McIlroy W.E., Staines W.R. (2014). The influence of an acute bout of aerobic exercise on cortical contributions to motor preparation and execution. Physiol. Rep..

[20] O’Leary K.C., Pontifex M.B., Scudder M.R., Brown M.L., Hillman C.H. (2011). The effects of single bouts of aerobic exercise, exergaming, and videogame play on cognitive control. Clin. Neurophysiol..

[21] Ferré S., O’Brien M.C. (2011). Alcohol and caffeine: The perfect storm. J. Caffeine Res..

[22] World Medical Association. Declaration of Helsinki. Ethical Principles for Medical Research Involving Human Subjects.

[23] Kaseda Y., Jiang C., Kurokawa K., Mimori Y., Nakamura S. (1998). Objective evaluation of fatigue by event-related potentials. J. Neurol. Sci..

[24] Borg G. (1970). Perceived exertion as an indicator of somatic stress. Scand. J. Rehabil. Med..

[25] Makudi W., Eugenio F. (2009). Futsal Official Rule Book. FIFA [Internet]. http://www.tff.org/Resources/TFF/Documents/TFF/MHK%20Talimatlar/Futsal-Oyun-Kural-Kitabi.pdf.

[26] Foster C., Florhaug J.A., Franklin J., Gottschall L., Hrovatin L.A., Parker S., Doleshal P., Dodge C. (2001). A new approach to monitoring exercise training. J. Strength Cond. Res..

[27] Yanci J., Martínez-Santos R., Los Arcos A. (2014). Respiratory and muscular perceived efforts after official games in professional soccer players. J. Strength Cond. Res..

[28] Ila A.B., Polich J. (1999). P300 and response time from a manual Stroop task. Clin. Neurophysiol..

[29] Verburgh L., Scherder E.J., van Lange P.A., Oosterlaan J. (2014). Executive functioning in highly talented soccer players. PLoS ONE.

[30] Huertas F., Zahonero J., Sanabria D., Lupiáñez J. (2011). Functioning of the attentional networks at rest vs. during acute bouts of aerobic exercise. J. Sport Exerc. Psychol..

[31] Hockey G.R.J. (1997). Compensatory control in the regulation of human performance under stress and high workload: A cognitive-energetical framework. Biol. Psychol..

[32] Paas F., Tuovinen J.E., Tabbers H., Van Gerven P.W. (2003). Cognitive load measurement as a means to advance cognitive load theory. Educ. Psychol..

[33] Pesce C., Tessitore A., Casella R., Pirritano M., Capranica L. (2007). Focusing of visual attention at rest and during physical exercise in soccer players. J. Sports Sci..

